# Advances in synthetic lethality modalities for glioblastoma multiforme

**DOI:** 10.1515/med-2024-0981

**Published:** 2024-06-10

**Authors:** Seidu A. Richard

**Affiliations:** Department of Medicine, Princefield University, P. O. Box MA128, Volta Region, Ho, Ghana; Institute of Neuroscience, Third Affiliated Hospital, Zhengzhou University, Zhengzhou, 450052, China

**Keywords:** GBM, DNA, chemotherapy, synthetic, lethality, resistance

## Abstract

Glioblastoma multiforme (GBM) is characterized by a high mortality rate, high resistance to cytotoxic chemotherapy, and radiotherapy due to its highly aggressive nature. The pathophysiology of GBM is characterized by multifarious genetic abrasions that deactivate tumor suppressor genes, induce transforming genes, and over-secretion of pro-survival genes, resulting in oncogene sustainability. Synthetic lethality is a destructive process in which the episode of a single genetic consequence is tolerable for cell survival, while co-episodes of multiple genetic consequences lead to cell death. This targeted drug approach, centered on the genetic concept of synthetic lethality, is often selective for DNA repair-deficient GBM cells with restricted toxicity to normal tissues. DNA repair pathways are key modalities in the generation, treatment, and drug resistance of cancers, as DNA damage plays a dual role as a creator of oncogenic mutations and a facilitator of cytotoxic genomic instability. Although several research advances have been made in synthetic lethality modalities for GBM therapy, no review article has summarized these therapeutic modalities. Thus, this review focuses on the innovative advances in synthetic lethality modalities for GBM therapy.

## 1 Introduction

Glioblastoma multiforme (GBM) is characterized by a high mortality rate, high resistance to cytotoxic chemotherapy, and radiotherapy due to its highly aggressive nature [1,2]. Also, it is the most typical primary malignant brain cancer, with no satisfactory surgical curability [1,2,3]. Furthermore, the prognosis of patients with GBM is often less than 1 year, notwithstanding modern innovative therapeutic modalities [1,4]. The pathophysiology of GBM is characterized by multifarious genetic abrasions that deactivate tumor suppressor genes, induce transforming genes, and oversecrete pro-survival genes, resulting in oncogene sustainability [5,6,7,8,9].

Most recently, targeted small molecule treatment modalities to block stimulated oncogenes with the aim of blocking several oncogenic pathways to synergize and destroy tumor cells have been ongoing [10]. Nevertheless, targeted small-molecule blockers are limited to a subset of all possible targets, called the “druggable genome,” and thus restrict the existing interruptible synergistic pathways [11,12]. Furthermore, recurrent tumors treated with targeted small molecules typically selected for targeted gene secondary-site mutations result in drug resistance due to mutator phenotypes [10,13]. Therefore, it is necessary to engineer selective anti-GBM treatment modalities based on GBM genetics that stimulate a synthetic lethal response that can change as rapidly as the GBM genetic landscape changes.

It is worth noting that cancer cells are imperiled to DNA damage and, more specifically, damage from double-strand breaks (DSBs), similar to normal cells [1,14]. Also, cells induce the DNA damage response (DDR) network as a feedback reaction to DNA damage, permitting DNA repair via the modulation of cell cycle succession, DNA damage repair, or apoptosis [1,15]. Synthetic lethality is a destructive process in which the episode of a single genetic consequence is tolerable for cell survival, while co-episodes of multiple genetic consequences lead to cell death [1,16]. This targeted drug approach, centered on the genetic concept of synthetic lethality, is often selective for DNA repair-deficient GBM cells with restricted toxicity to normal tissues [17,18].

Remarkably, DNA repair pathways are key modalities in the generation, treatment, and drug resistance of cancers, as DNA damage plays a dual role, such as a creator of oncogenic mutations and a facilitator of cytotoxic genomic instability [17,19]. Additionally, gene paralogs are potentially auspicious bases for synthetic lethal interactions, as they typically demonstrate robust sequence homology and functional redundancy. Although several research advances have been made in synthetic lethality modalities in GBM therapy, no review article has summarized these therapeutic modalities. Therefore, this review focuses on innovative advances in synthetic lethality modalities in glioblastoma therapy.

**Figure 1:**
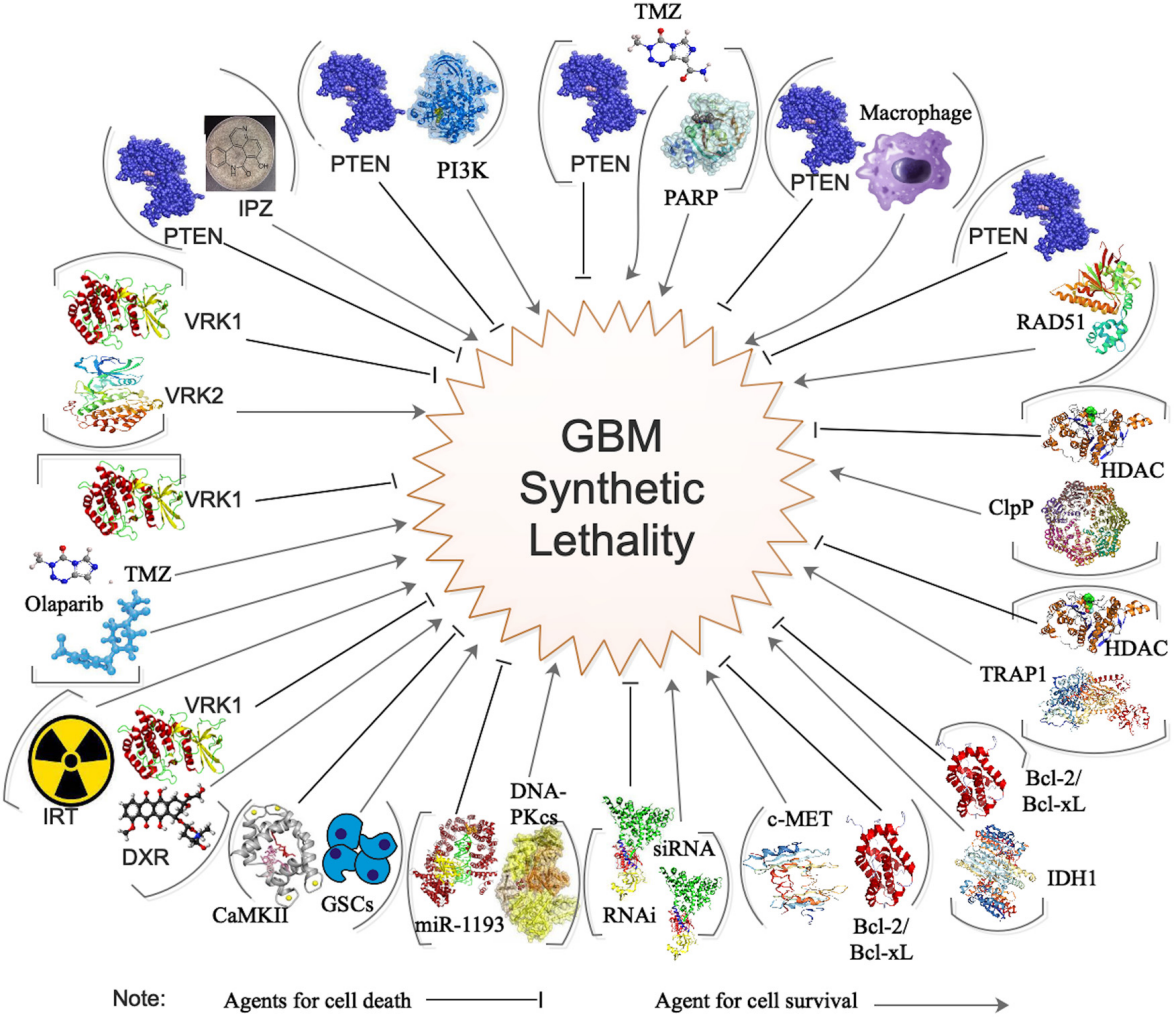
Combinations of literature search teams. It also shows the potential pairing of agents for synthetic lethality modalities in GBM therapy. Note: Refer to the text for detailed explanations. Also, refer to general abbreviation list for meaning of abbreviations.

The “Boolean logic” was used to search for articles on synthetic lethality modalities for GBM in PubMed and PubMed central as well as google scholar. Notably, only preclinical studies of synthetic lethality modalities for GBM have been reviewed. Also, clinical studies were excluded from this review. The combinations of the literature search teams are shown in Figure 1. This figure also shows the detailed potential pairing of agents for synthetic lethality modalities in GBM therapy.

## 2 Phosphatase and tension homologue (PTEN)

Loss of PTEN has been implicated in chromosomal instability, susceptibility to DNA damaging agents and compromised genomic integrity [20,21,22]. PETN has also been associated with DNA DSB repair [22,23]. Moreover, DNA DSBs are the most lethal form of DNA damage, and they are repaired by two key PETN pathways: non-homologous end-joining (NHEJ) and homology-directed repair (HDR) [20]. Also, PTEN deletion on chromosome 10 adversely stimulates the phosphoinositide 3-kinases (PI3K)/protein kinase B (AKT)/rapamycin (mTOR) signaling pathway, resulting in cellular differentiation, proliferation, and apoptosis [17,20].

However, mutated or deleted PTEN has been associated with resistance to radiotherapy and chemotherapy GBM patients [17,24,25]. In addition, nuclear PTEN is an essential factor in the maintenance of genomic stability because its loss is linked to chromosomal aberrations and impaired DSB repair capability [26,27]. Thus, PTEN’s function in various cellular processes, including the DDR, makes its deficiency in GBM a promising target for synthetic lethality therapeutic strategies [28,29,30].

### 2.1 PTEN and PI3Ks

In GBM, the stimulation of PI3K occurred autonomously with the upregulation of epidermal growth factor receptor (EGFR) via gain-of-function mutations in the structural gene for PI3Kα (PIK3CA) and by the loss of PTEN, a downregulator of PI3K signaling (Table 1) [31,32]. It was observed that blockade of PI3K efficiently inhibited survival signaling pathways via AKT, whereas blockade of PI3K alone and in combination with blockade of EGFR and of the down-regulatory kinase mTOR failed to stimulate cell death *in vitro* or *in vivo* in preclinical schemes (Table 1) [33,34].

**Table 1:** Summarizes cell death agents and cell survival agents and their mechanism of synthetic lethality in GBM

Agents for cell death	Agent for cell survival	Synthetic lethality mechanisms in GBM	Citation
PTEN	PI3K	Stimulation of PI3K occurred autonomously with upregulation of EGFR via gain-of-function mutations in the structural gene for PI3Kα (PIK3CA) and by loss of PTEN in GBM	[31,32]
		Blockade of PI3K efficiently inhibited survival signaling pathways via AKT, implying that P13K is key for survival of GBM	[33,34]
	PARP & TMZ	PARP-1 blockade and PTEN deficiency offered synthetic lethality in TMZ cells deficient in MGMT and MGMT‑inhibited cells	[45]
		The 3-combination triggered G2/M arrest because of the generation of DSBs, perhaps via the alteration of N-methylpurine abrasions, which were not removed because of repair intervention by PARPi, leading to the induction of apoptosis in GBM	[45]
	Irrepairzepine	It stimulated synthetic lethality in the genetic architecture of PTEN loss due to its exhibited capacities of inhibiting DSB repair in GBM	[17]
		Irrepairzepine augmented irradiation cytotoxic consequences with a reduction in cell survival detected in the PTEN-deficient cells as compared to only radiotherapy	[17]
	Symbiotic Macrophage	PTEN inactivation induced the stimulation of multiple down-regulatory signaling pathways such as AKT and SRC in GBM cells, and LOX blockade impaired GBM progression via the inhibition of macrophage infiltration without directly influencing GBM cells	[49,52]
		LOX expression from PTEN-deficient GBM cells and incorporation into macrophages is a continuous process that is capable of stimulating PYK2 signaling, and lysosomal degradation may influence LOX-regulated down-regulatory pathways	[49]
	RAD51	PTEN/RAD51 signaling axis functions in response to replication stress to guarantee effective DNA replication because loss of PTEN resulted in replication stress in GBM	[54]
		3E10, a RAD51 facilitator, influenced the cellular viability of PTEN-deficient cells, and blockade of HDR with 3E10 triggered cytotoxicity in PTEN-deficient cells in GBM	[20]
		GBM cells deficient in PTEN are sensitive to the combination of 3E10 and an ATR inhibitor (VE-822)	[20]
VRK1	VRK2	VRK1 is a paralog synthetic-lethal target in VRK2-methylated GBM because silencing of VRK1 in VRK2-null and VRK2-low secreting GBM cell lines is lethal, and leads to defective nuclear envelope formation, G2-M arrest, and consequently DNA damage	[56]
	TMZ & Olaparib	Depletion of VRK1 in combination with TMZ and olaparib in GBM cells triggered an upsurge in DNA damage at lower doses, which led to the death of tumor cells	[38,79,42]
	Radiation & DXR	VRK1 depletion triggered synthetic lethality in combination with IRT or DXR, and VRK1 depletion resulted in a significant reduction in the dose needed to achieve a similar effect in GBM	[67,80]
HDAC1/2	ClpP	HDAC inhibitors and imipridones have opposite effects on oxidative metabolism, while HDAC inhibitors and imipridones inhibit tumor cell’s respiration in GBM	[85]
		Imipridones suppress the secretion of respiratory complexes in an approach mainly determined by the ClpP protease, whereas HDAC inhibitors transcriptionally upregulate enzymes and transcription factors that accelerate oxidative metabolism in GBM	[85]
	TRAP1	Gamitrinib and its target TRAP1 were capable of thwarting HDAC inhibitor-triggered stimulation of tumor respiration via disruption of the electron transport chain in GBM	[84,100]
Bcl-2/Bcl-xL	IDH1	Blockade of Bcl-xL triggers synthetic lethality in IDH1-mutated GBM cells *in vitro* and *in vivo,* and their influences are mediated by the oncometabolite, 2-HG, which decreases Mcl-1 protein concentrations	[103]
		IDH1 R132H facilitated the inhibitory effects of Bcl-xL either via siRNA or pharmacologically via the BH3-mimetic, ABT263 in tumor cells	[103]
	c-MET	c-MET blockade, along with dual Bcl-2/Bcl-xL blockade, triggered synthetic lethality in stem-like and established GBM cells, which was pharmacologically epitomized by the drug combination of ABT263 and Crizotinib	[101]
siRNA	RNAi	siRNAs directly targeted truncated EGFRvIII and AKT2 and synergized to stimulate an apoptotic synthetic lethal RNAi response in an orthotopic GBM xenograft mouse model	[16,117]
CaMKII	GSCs	A synthetic curcumin derivative HBC, a Ca^2+^/CaM antagonist, blocked not only the self-renewal capacity but also the metastatic potential of GSCs by inhibiting the CaM/CaMKII/c-Met signaling pathway	[123,128]
		NK1R blockers, such as SR 140333 and aprepitant, have substantial chemical synthetic lethal activity against CaMKII blockers, such as HBC, berbamine, and KN93, in GSCs	[123]
		A synthetic lethal association between CaMKII and NK1R via RNAi in GSCs and the synergistic therapeutic effect of the combination of CaMKII and NK1R blockers on GSCs was linked to the downregulation of PI3K/AKT/NF-κB and Ca^2+^-signaling in GBM	[123]
		The lethal consequence of CaMKII and NK1R blockers on GSCs was correlated with a significant decrease in the secretion of key GSC markers, such as ALDH1A1, CD44, CD133, Oct4, Sox2, and integrin α6 in GBM	[123]
		The synergistic anti-GBM consequence of CaMKII and NK1R blockers against GSCs was triggered by the facilitation of ROS-dependent apoptosis via robust stimulation of the caspase cascade intermediated by p53	[123]
miR-1193	DNA-PKcs	Perturbation of both DSB repair pathways like HR/MMEJ and NHEJ via concurrent blockade of miR-1193 and ablation of DNA-PKcs is capable of triggering obvious loss of viability in GBM	[1]
		Concurrent blockade of these distinctive DSB repair pathways resulted in the stimulation of the ATR/CHK1/p53 axis and consequently facilitated tumor cell apoptosis	[1]
		Silencing of DNA-PKcs and the blockade of miR-1193 in GBM is a potential synthetic lethality target for patients with GBM	[1]

Note: refer to general abbreviation list for the meaning of abbreviations.

The imidazopyridine blocker PIK-75 is capable of stimulating apoptosis rather than growth arrest in gliomas [31]. However, the broad target profile and comprehensive toxicity of PIK-75 have hindered its clinical development because of its lack of stability in solution and *in vivo* [35,36]. Nevertheless, using a PI3Kα-specific blocker in amalgamation with a clinical blocker of cyclin-dependent kinase (CDK)-1 and CDK2, the apoptotic effects of the parental compound were reiterated, resulting in the development of an amalgamated therapy that exhibited decreased toxicity and stimulated apoptosis *in vivo* in murine xenografts [31]. Further studies on synthetic lethality modalities using PIK-75 and PTEN are needed.

### 2.2 PTEN, PARP, and temozolomide (TMZ)

Poly (ADP-ribose) polymerase (PARP) inhibitors are able to overcome limitations due to cancer-driving breast cancer gene (BRCA) mutations via the blockade of compensatory repair pathways that BRCA-deficient cancer cells depend on for survival because of their dysfunctional homologous recombination-mediated DSB repair [30,37,23]. In addition, PARP inhibitors and DNA damage in ataxia-telangiectasia mutated (ATM) signaling protein kinases have been shown to eliminate PTEN-deficient cancer cells in different tumor types [38,39,40]. TMZ is an alkylating agent that alters DNA, resulting in 06-meG, N3-meG, N7-meG, and N3-meA abrasions [41,42]. Also, resistance to TMZ therapy arises in GBM cells secreting high concentrations of O-6-methylguanine-DNA methyltransferase (MGMT), an enzyme that directly deletes the methyl group added by TMZ to O6-mG [43,44].

Notably, GBM cells have a high DDR, which allows them to repair abrasions caused by TMZ [43,44]. MGMT is a key gene that triggers TMZ resistance during GBM chemotherapy [45]. Many studies have focused on the influence of PTEN deficiency on repair pathways and the effects of PARP-1 blockade and PTEN deficiency in terms of synthetic lethality in TMZ-treated GBM cells [46,47,48]. It was observed that the combined treatment with TMZ and NU1025 was efficient in decreasing cell viability and clonogenic survival rates exhibited by TMZ-resistant cells, such as T98G and LN18, which are MGMT-proficient cells (Table 1) [45]. Also, a potentiating effect was observed when TMZ and PARP inhibitor (PARPi) were combined, which was independent of MGMT activity in U251MG sensitive-TMZ cells deficient in MGMT and MGMT‑inhibited cells in T98G and LN18 cell lines (Table 1) [45].

Interestingly, this drug combination triggered G2/M arrest because of the generation of DSBs, perhaps via alteration of N-methylpurine abrasions, which were not removed because of repair intervention by PARPi, leading to the induction of apoptosis in T98G and LN18 cells [45]. Moreover, the reduction in viability of U87MG cells transpired as a result of TMZ-induced DSBs in the combined therapies [45]. In these cells, the augmented quantity of unrepaired DNA damage was initiated by the blockade of PARP-1 via NU1025, resulting in cell death following combined drug treatments [45]. On the other hand, the use of PARPi, such as GPI 15427, in which MGMT enzyme activity was absent, revealed contrary results in TMZ-sensitive U87MG cells [46].

### 2.3 PTEN and irrepairzepine (IPZ)

A crude fungal isolate, E14504F, exhibited selective cytotoxicity in PTEN-deficient GBM cells compared to isogenic PTEN-proficient cells, with a 33% difference in survival [17]. It was established that the endophyte generated a compound that is active in the cellular architecture made up of PTEN loss, which was established by the absence of activities in cell lines mutated in the tumor-associated genes, such as Kirsten rat sarcoma viral oncogene homologue and BRCA2 [17].

Furthermore, activity-guided structural classification showed that the endophyte generated an azepine core-containing alkaloid called IPZ [17]. This novel molecule stimulated synthetic lethality in the genetic architecture of PTEN loss owing to its ability to inhibit DSB repair in GBM (Table 1) [17]. In addition, in a cell viability assay, E14504F exhibited dose-dependent cytotoxicity in U251 cells, with synthetic lethality in PTEN-null cells persisting across all tested doses [17]. Thus, IPZ can potentially act as an anticancer molecule for the further development and treatment of PTEN-deficient GBMs [17].

It was observed that IPZ treatment alone was adequate in the stimulation of appreciably more DNA damage in the PTEN-deficient cells compared to PTEN-proficient cells, signifying potential blockade in the repair of intuitively occurring DSBs and IPZ selectively suppressed DSB repair in GBM (Table 1) [17]. Furthermore, IPZ treatment augmented irradiation cytotoxic consequences with a reduction in cell survival detected in the PTEN-deficient cells compared to only radiotherapy (IRT) (Table 1) [17]. Thus, IPZ’s capability of significantly eradicating PTEN-deficient GBM cells, in addition to sensitizing these cells to IRT-induced DNA damage, makes it a possible drug for combination with IRT [17].

### 2.4 PTEN and symbiotic macrophage

It has been established that PTEN deficiency facilitated yes-associate protein (YAP)-1-driven lysyl oxidase (LOX) secretion in GBM cells and that LOX stimulated the β1 integrin-proline-rich tyrosine kinase (PYK)-2 pathway in macrophages to facilitate their infiltration into the GBM tumor microenvironment (TME) [49]. Also, tumor-associated microphages (TAMs) are known to facilitate GBM cell survival and angiogenesis in addition to influencing the function of other immune cells in the GBM TME [50,51].

Remarkably, PTEN inactivation induces the stimulation of multiple downstream signaling pathways, such as AKT and SRC, in GBM cells (Table 1) [49,52]. Moreover, LOX blockade impairs GBM progression by inhibiting macrophage infiltration without directly influencing GBM cells (Table 1) [49]. Also, macrophage β1 integrin was capable of facilitating LOX uptake and drove the LOX responsiveness of macrophages in GBM [49]. Furthermore, PYK2, but not focal adhesion kinase (FAK), is required for LOX-stimulated macrophage infiltration in GBM [49].

Interestingly, LOX expression in PTEN-deficient GBM cells and its incorporation into macrophages is a continuous process that is capable of stimulating PYK2 signaling, and lysosomal degradation may influence LOX-regulated downregulatory pathways (Table 1) [49]. Thus, LOX precisely recruits macrophages to GBM via the β1 integrin-PYK2 pathway [49]. Furthermore, LOX blockade augmented the survival of PTEN-deficient GBM models, coupled with the fact that the LOX blocker is BBB-penetrable, which should influence the testing of β-aminopropionitrile, precisely in PTEN-deficient GBM patients [49].

### 2.5 PTEN and anti-RAD51 antibody

RAD51 is a crucial stimulus in stalled replication forks and the repair of DNA breaks in collapsed forks [20,53]. Also, XRCC3 and RAD51-mediated strand invasion can trigger fork restart if stalled replication forks are undamaged [53]. Furthermore, a new source of firing is often obligatory to rescue replication in situations of collapsed replication forks, and repair of collapsed forks is determined by classical RAD51-mediated HDR [53]. It has been established that the PTEN/RAD51 signaling axis functions in response to replication stress to guarantee effective DNA replication because loss of PTEN results in replication stress (Table 1) [54]. Also, 3E10, a RAD51 facilitator, influences the viability of PTEN-deficient cells and blockade of HDR with 3E10-triggered cytotoxicity in PTEN-deficient cells (Table 1) [20].

Interestingly, cytotoxicity occurred because PTEN-deficient cells are both deficient in NEHJ and have a substantial standard burden of DNA damage and replication stress that was aggravated by 3E10 treatment, as shown by the stimulation of ɣH2Ax and p53BP1 foci and the manifestation of micronuclei after 3E10 scFv treatment [20,27]. Also, the cellular and replicative stresses triggered by 3E10 in PTEN-deficient cells sensitize these cells to a small molecule inhibitor of ATM- and Rad3-related (ATR) kinase [20]. ATR kinase is recruited to replicate protein A (RPA)-coated single-stranded DNA at stalled replication forks and sites of DNA damage [55]. Thus, cells deficient in PTEN are sensitive to a combination of 3E10 and an ATR inhibitor (VE-822) (Table 1) [20].

## 3 Vaccinia-related kinases (VRKs)

VRKs are a group of serine/threonine kinases that participate in the regulation of transcription factors, chromatin transformation, nuclear envelope configuration, and cell-cycle succession [56,57]. This group consists of three members: VRK1, VRK2, and pseudo-kinase VRK3 [56,57]. VRK1 is localized in both the nucleus and cytosol, while VRK2 is localized to the endomembrane of the endoplasmic reticulum and the nuclear envelope [57]. VRK1 is associated with phosphorylation and multiple substrates in both cell-cycle progression and cell-cycle arrest [56,57].

Specifically, VRK1 phosphorylates histones H3 and H2AX to accelerate chromatin remodeling, transcription factors ATF2, CREB, Sox2, and farnesoid X nuclear receptor HR1H4 to stimulate cell cycle succession, and a barrier to autointegration factor (BAF) to modulate nuclear envelope formation in response to mitogenic stimuli [58,59]. Also, VRK1 phosphorylates p53, c-Jun, and 53BP1 to induce cell-cycle arrest for DNA damage repair in response to stress signals such as DNA damage [58,59].

However, the precise functional role of VRK2 remains a matter of debate. Nevertheless, it has been implicated in the downregulation of apoptosis via direct interaction with antiapoptotic proteins, such as B-cell lymphoma-extra large (Bcl-xL), and via downregulation of proapoptotic protein like B-cell lymphoma 2 (Bcl-2)-associated X protein [60]. Furthermore, p53 and BAF have been implicated as substrates for VRK2 [61]. Also, cells with low VRK2 secretion exhibit augmented sensitivity to chemotherapeutics [60].

Notably, VRK1 is one of the most abundant nuclear kinases in human cells, and its over-secretion is related to poor outcomes in GBM and neuroblastoma [62,63,64]. Moreover, VRK1 has been implicated as a potential new cancer treatment target in the context of cancer dependency after several CRISPR-enabled functional genomic screening experiments [65,66]. VRK1 depletion is beneficial for cells deficient in ATM or p53, which widens the number of tumor categories and may be used as an effective therapy [67,68].

### 3.1 VRK1 and VRK2

Notably, VRK1 was identified as a paralog synthetic-lethal target in VRK2-methylated GBM and neuroblastoma cell lines [56]. They indicated that silencing of VRK1 in VRK2-null and VRK2-low secreting GBM cell lines is lethal and leads to defective nuclear envelope formation, G2-M arrest, and consequent DNA damage (Table 1) [56]. Their synthetic-lethal association was repeated *in vivo* in a VRK2-methylated U251MG GBM xenograft prototype, where VRK1 silencing resulted in tumor regression [56]. They further established that the enzymatic activity of VRK1 was a prerequisite for VRK1–VRK2 synthetic lethality, which specifies a path for small-molecule drug discovery [56].

Therefore, VRK1 is a promising synthetic-lethal drug target for VRK2-methylated brain tumors [56]. Initial studies have demonstrated that VRK1 modulates the cell cycle with roles in G1-S progression as well as mitosis [69,70,71,72,73]. Also, VRK1 depletion led to a preserved interaction of nuclear envelope fragments with mitotic chromosomes, resulting in aberrant nuclear envelope reassembly, nuclear bridging between daughter cells, and eventually DNA damage and apoptotic cell death [74].

### 3.2 VRK1, TMZ, and Olaparib

Olaparib is a selective and effective inhibitor of PARP enzymes such as PARP1 and PARP2 [42,67]. Olaparib targets the base excision repair (BER) pathway, which repairs alkylating lesions in the DNA [42,75]. Olaparib is a therapeutic agent for tumors that already have modifications in some DDR pathways, such as BRCA1, BRCA2, or WRN mutations [76,77,78]. Studies have shown that VRK1 depletion sensitizes tumor cells to TMZ and olaparib (Table 1) [38,79]. Depletion of VRK1 in combination with TMZ and olaparib in GBM cells triggered an upsurge in DNA damage at lower doses, which led to the death of tumor cells (Table 1) [42]. Thus, targeting VRK1 may be a potential therapeutic option when precise VRK1 inhibitors are developed [42]. The combined use of olaparib and VRK1 inhibitors has potential as a cancer therapeutic regimen with less severe or fewer adverse effects and toxicity with better tolerance [42].

### 3.3 VRK1, radiation, and doxorubicin (DXR)

Synthetic lethality of VRK1 depletion was observed when used in combination with other DNA damage treatments, as previously described [67]. Studies have shown that VRK1 depletion triggered synthetic lethality in combination with radiation (IRT) or DXR, and VRK1 depletion resulted in a significant reduction in the dose needed to achieve a similar effect in GBM (Table 1) [67,80]. Pyrimidine-based inhibitors have also exhibited high affinity and specificity for the VRK1 kinase, which can be a candidate for future drug development [81]. Further studies on the synthetic lethality of VRK1, IRT, and TMZ are required.

## 4 Histone deacetylase

Global and selective HDAC inhibitors are capable of disrupting the Warburg effect by targeting super-enhancers of genes such as HK2, GAPDH, and ENO1 [82,83]. Studies have observed that partial cell eradication and the survival of GBM cells resulted in a decrease in glycolytic activity; in order to sustain survival, they accelerated tumor respiration following treatment with HDAC inhibitors [84,82]. Nguyen et al. demonstrated that global (panobinostat) and selective (romidepsin) HDAC inhibitors combined with gamitrinib synergistically decreased the viability of a broad diversity of GBM models [84]. Nguyen et al. demonstrated that specific stimulation of the Y118A is synthetically lethal with either HDAC1 or HDAC2 blockade via the stimulation of intrinsic apoptosis based on both antiapoptotic Bcl-xL and Mcl-1 GBM models [85].

Furthermore, dopamine receptors were discovered as targets for imipridones, and subsequently, high concentrations of dopamine receptors DRD2 bestowed augmented susceptibility of cancer cells to the cytotoxic effects of imipridones [86]. Studies have also implicated imipridones in mitochondrial energy metabolism because ONC201, ONC206, and ONC212 interfere with the oxygen consumption rate (OCR), mitochondrial respiration, and coupled respiration [85,87]. Moreover, imipridones regulate glycolysis in a cell type- and context-dependent manner [87]. Studies have demonstrated that imipridone-mediated upregulation of activating transcription factor 4 (ATF4) partially induces the serine glycine synthesis pathway by stimulating PHGDH, PSAT1, and PSPH, along with the inhibition of tumor energy metabolism [87,88].

### 4.1 HDAC1/2 and caseinolytic protease proteolytic (ClpP)

Notably, GBMs have critical metabolic dependencies, such as carbohydrates, amino acids, and fatty acid metabolism [89,90,91,92]. Thus, GBMs have a central core that typically depends on glycolysis, which is enclosed by a periphery determined by oxidative metabolisms, such as the beta oxidation of fatty acids [93,85]. Interestingly, the ClpP protein can cleave respiratory complexes associated with metabolism, resulting in cellular respiration and ATP production via oxidative phosphorylation upon stimulation [94,95,96].

Remarkably, the ClpP protease (Y118A) mutant was primarily active, whereas ClpP (D190A) rendered the enzyme inactive [94,95,96]. Also, this combination therapy exerts its influence partly by inhibiting oxidative energy metabolism in an approach strongly determined by ClpP protease because the ClpP-D190A mutant abolished the killing effects and the metabolic effects of the combination therapy[85]. Notably, these compounds are effective stimulators of the death ligand TRAIL [97,98]. However, it is now clear that imipridones (i.e., ONC201) have anti-tumor effects by blocking the proliferation or regulation of intrinsic apoptosis via suppression of Mcl-1 [99,85]. Also, ClpP is a new target for imipridones and is associated with ClpP protease in tumor respiration and oxidative phosphorylation [99,94,95,96].

Furthermore, these compounds are capable of stimulating integrated stress response with an obvious increase in ATF4 [85]. Interestingly, HDAC inhibitors and imipridones have opposite effects on oxidative metabolism, while HDAC inhibitors drive imipridones to inhibit tumor cell respiration (Table 1) [85]. Also, imipridones suppress the secretion of respiratory complexes in an approach mainly determined by the ClpP protease, whereas HDAC inhibitors transcriptionally upregulate enzymes and transcription factors that accelerate oxidative metabolism (Table 1) [85]. Thus, panobinostat may be successfully combined with clinically certified imipridones such as ONC201, ONC206, and ONC212 [85].

### 4.2 HDAC1/2 and TNF receptor-associated protein 1 (TRAP1)

Interestingly, gefitinib and its target TRAP1 were capable of thwarting HDAC inhibitor-triggered stimulation of tumor respiration, asgamitrinib was conspicuously recognized as a disruptor of the electron transport chain in GBM (Table 1) [84,100]. Thus, these drug combinations, including gamitrinib and HDAC inhibitors, seem to be efficient against a wide variety of GBM models, signifying their potentially broad applicability, specifically with regard to heterogeneity [84]. Furthermore, the combined blockade of TRAP1 and HDACs is a potential novel strategy to combat recalcitrant GBM [84].

## 5 Bcl-2

Bcl-2 proteins are key regulators with pro- and antiapoptotic functions. Notably, the antiapoptotic Bcl-2 sub-groups are inhibited by selective compounds called Bcl-2 homology domain 3 (BH3)-mimetics, which elicit on-target efficiency in the nanomole range [101,102]. BH3-mimetics have been utilized in numerous preclinical and clinical drug combination treatment regimes because they are elegant and a new approach to tackling tumor cells [102]. In contrast, antiapoptotic Bcl-2 sub-groups, such as Bcl-xL and myeloid cell leukemia-1 (Mcl-1), are highly secreted in human GBM and have been implicated in substantial anti-GBM activity [103]. However, the disadvantage of the blockade of Bcl-xL is that the function of platelets is determined by Bcl-xL, and extensive blockade of Bcl-xL may trigger thrombocytopenia [104]. Interestingly, short interfering RNA (siRNA) analyses revealed that Noxa was associated with cell death triggered by Bcl-2/Bcl-xL blockade and 2-HG treatment, signifying that Noxa is required in the death and not a “bystander” effect [103].

### 5.1 IDH1 and Bcl-xL

Mutations in isocitrate dehydrogenase (IDH)-1 and, to a lesser extent, in IDH2 predict appreciably enhanced overall survival in comparison with wild-type IDH GBMs and IDH-mutant GBMs [103,105]. Also, whereas IDH1 resides in the cytoplasm, IDH2 and IDH3 are localized to the mitochondria [105]. Furthermore, IDH1 and IDH2 catalyze irreversible reactions, resulting in the generation of NADPH2 and alpha-ketoglutarate, whereas IDH3 often reversibly utilizes NADH2 [103,105]. Moreover, NADPH2 is capable of facilitating an anabolic state in cancer cells by delivering electrons for certain steps in metabolic synthesis, such as fatty acid and nucleotide synthesis, such as ribonucleotide reductase [103,105].

In IDH1-mutated GBM, NADPH2 functions as a cofactor for the reduction of alpha-ketoglutarate to 2-HG and 2-HG at substantial concentrations (30 mM) [103]. Furthermore, 2-HG disrupts the normal function of dioxygenases that utilize alpha-ketoglutarate as a cofactor because of its structural similarity to alpha-ketoglutarate [106]. Moreover, these dioxygenases are particularly relevant in histone and DNA demethylation, which trigger a hypermethylated phenotype in IDH1-mutated tumors [107]. Notably, blockade of Bcl-xL triggers synthetic lethality in IDH1-mutated GBM cells *in vitro* and *in vivo,* and their effects are mediated by the oncometabolite 2-HG, which decreases Mcl-1 protein concentration (Table 1) [103]. Markedly, it was detected that IDH1 R132H facilitated the inhibitory effects of Bcl-xL either via siRNA or pharmacologically via the BH3-mimetic ABT263 in tumor cells (Table 1) [103].

Nevertheless, IDH1-mutated GBM and engineered IDH1-mutated GBM cells exhibited lower concentrations of Mcl-1 and treatment with a cell-permeable form of 2-HG inhibited Mcl-1 protein concentration in IDH1 wild-type cells [103]. Also, the 2-HG-mediated decrease in Mcl-1 protein concentration was associated with less protein synthesis and a partial shutdown of mTORC1 signaling, which supports the fact that 2-HG lengthens the lifespan of C. elegans in part by blocking mTORC1 signaling [108,109]. Furthermore, both mutant IDH1- and 2-HG-treated cells exhibited lower baseline OCRs and ATP levels, which in part mediated a decrease in protein synthesis, mTORC1 signaling, and ultimately a reduction in Mcl-1 concentrations [103].

### 5.2 Bcl-2/Bcl-xL and c-MET

c-Mesenchymal-epithelial transition factor (c-MET) is essential for the growth and maintenance of stem-like GBM cells, a group of tumor cells within glial brain tumors responsible for therapeutic resistance and progression [101,110,111]. Remarkably, Mcl-1 blocks mitochondrial outer membrane permeabilization and release of cytochrome-c into the cytosol after binding to BCL-2 antagonist/killer (BAK) [101]. Also, Mcl-1 absorbs BAK to counteract cell death by inhibiting Bcl-xL and Bcl-2, and Mcl-1 is pivotal for the survival of cancer cells [101]. Furthermore, Mcl-1 is localized in the mitochondrial matrix, directly regulates OXPHOS, and aids in driving oxygen-determined ATP generation [112].

Moreover, GBM sphere cultures exhibited augmented phosphorylation of c-MET, which mediates transcription factor modulation reprogramming essential for a “stem-cell” state [101]. Notably, ectopic secretion of c-MET blocks the differentiation of stem-like GBM cells in a Nanog-dependent fashion, affirming the pivotal function of this kinase pathway in GBM stem cells [111]. Additionally, c-MET modulates the secretory levels of Mcl-1 protein in human GBM models, and GBM stem-like cells exhibit elevated concentrations of c-MET [101].

Interestingly, an important association between c-MET signaling and Mcl-1 in this essential cell population specified that interference with c-MET is a new target for the suppression of Mcl-1 concentrations [101]. Remarkably, c-MET blockade, along with dual Bcl-2/Bcl-xL blockade, triggered synthetic lethality in stem-like and established GBM cells, which were pharmacologically epitomized by the drug combination of ABT263 and Crizotinib, which are currently being tested and used in treating patients (Table 1) [101].

## 6 RNA interference (RNAi) and siRNA

siRNA is 20–25 nucleotide-long double-stranded RNA (dsRNA) molecules that are capable of selectively inhibiting specific genes via sequence-specific mRNA transcript mortification [113,114,115]. Studies have identified 16 genes that are functionally linked to cell death pathways. Also, the products of these genes function as nuclear hormone receptors such as GTPases, G-protein coupled receptors, oxidases, and mutases, whereas numerous genes were uncategorized [113,116]. Notably, 9 of the 16 genes have been implicated in high secretory levels in GBM [116].

Studies have shown that RNAi is capable of detecting nonessential target genes via *in vitro* screening for synthetic lethality by means of siRNA/shRNA libraries such as small-molecule compound libraries and, in parallel, using *in vitro* and *in vivo* models [16,117,118]. Also, an amalgamation of RNAi responses was capable of augmenting target knockdown efficiency, and up to ten independent mRNAs were potentially targeted by RNAi [117,119]. Furthermore, one siRNA screen-detected blockade of multiple kinases was robust in sensitizing CAL51 and HeLa cells to the DNA repair enzyme PARP inhibition [117,120].

Inhibition of CDK2 by RNAi resulted in the stimulation of synthetic lethality in NMYC-augmented neuroblastoma cell lines but not in single-copy NMYC neuroblastoma cell lines [16,117]. GBM is associated with hyperactivation of AKT and augmentation or truncated mutations of EGFR [121]. Experimentally, a peptide transduction delivery domain (PTD) fused to a dsRNA-binding domain (PTD-DRBD) was utilized to deliver siRNAs that directly targeted truncated EGFRvIII and AKT2 and synergized to stimulate an apoptotic synthetic lethal RNAi response in an orthotopic GBM xenograft mouse model (Table 1) [16,117].

Nevertheless, targeting EGFRvIII, AKT1, or AKT3 does not stimulate a synthetic lethal RNAi response [16]. However, a protein transduction domain derived from the HIV transactivator of transcription (TAT) transports siRNA into GBM cells to stimulate its RNAi effect[16]. In addition, an siRNA delivery method that uses the recognized macromolecular delivery potential of PTDs combined with the sequence-independent dsRNA binding avidity of DRBD allows for the transportation of all siRNA sequences [16].

## 7 CaMKII and GSCs

Calcium (Ca^2+^)/calmodulin (CaM)-dependent protein kinase II (CaMKII), one of the most critical modulators of Ca^2+^-signaling and multifunctional serine/threonine kinase [122,123]. Studies have demonstrated that CaMKII is very essential for the survival, proliferation, invasion, and differentiation of numerous cancer cells via the stimulation of multiple signaling pathways, such as the extracellular signal-regulated kinase, the signal transducer and activator of transcription 3 (STAT3), AKT, and Wnt/β-catenin signaling pathways [124,125]. GBM stem-like cells (GSCs) are produced by GBM and participate in tumor initiation, immune evasion, cancer invasion, radiotherapy, chemotherapy resistance, and recurrence [126,127].

Notably, a synthetic curcumin derivative, hydrazinobenzoylcurcumin (HBC), a Ca^2+^/CaM antagonist, blocked not only the self-renewal capacity, but also the metastatic potential of GSCs by inhibiting the CaM/CaMKII/c-Met signaling pathway [123,128]. Also, a selective CaMKII inhibitor, KN93, is capable of blocking the growth of GSCs and secretion of GSC stemness markers [128]. Furthermore, CaMKIIγ silencing reduced the stem-like characteristics of GBM cells [128].

Remarkably, neurokinin 1 receptor (NK1R) blockers, such as SR 140333 and aprepitant, have substantial synthetic lethal activity against CaMKII blockers, such as HBC, berbamine, and KN93, in GSCs (Table 1) [123]. Moreover, combined therapy with CaMKII and NK1R blockers not only reduced GSC viability but also potently suppressed GSC-derived tumor growth in an *in vivo* model of tumorigenesis [123]. Thus, there is a synthetic lethal association between CaMKII and NK1R via RNAi in GSCs [123]. Also, the synergistic therapeutic effect of the combination of CaMKII and NK1R blockers on GSCs was linked to the downregulation of PI3K/AKT/NF-κB and Ca^2+^signaling in GBM (Table 1) [123].

Furthermore, the lethal consequences of CaMKII and NK1R blockers on GSCs were correlated with a significant decrease in the secretion of key GSC markers such as ALDH1A1, CD44, CD133, Oct4, Sox2, and integrin α6 in GBM (Table 1) [123]. Specifically, the synergistic anti-GBM effect of CaMKII and NK1R blockers against GSCs was triggered by the facilitation of ROS-dependent apoptosis via robust stimulation of the caspase cascade mediated by p53 (Table 1) [123]. Furthermore, NK1R agonists, such as SP and hemokinin-1 (HK-1), facilitate GBM cell proliferation and migration, whereas NK1R antagonists, such as aprepitant, essentially block GBM cell growth *in vitro* and *in vivo* [129,130]. Thus, NK1R may function as an upstream modulator of CaMKIIγ activity[123].

## 8 DNA-PKcs and miR-1193

MicroRNAs (miRNAs or miRs) are small, and extremely preserved, noncoding RNA molecules that typically inhibit gene translation by attaching to complementary sequences in the 3′ untranslated regions (3′UTRs) of their target mRNAs [1,131]. Studies have shown that miRNAs are essential for the initiation, progression, and recurrence of human cancers [132,133,134]. In retinoblastoma formation, synthetic lethality between the miR-17-92 cluster and tumor suppressor p53 was discovered [135]. Synthetic lethality between miR-206 and c-Myc via direct blockade of MAP3K13 was also discovered [136].

Flap endonuclease1 (FEN1), a structure-specific endonuclease, has been implicated in DSB repair via HR and MMEJ and via the long patch BER (LP-BER) pathway[137,138]. FEN1 has also been implicated as a potential strategy for GBM therapy because silencing FEN1 triggered augmented cisplatin sensitivity in GBM [139]. In contrast, the DNA-dependent protein kinase catalytic subunit (DNA-PKcs) is essential for DSB repair via the NHEJ pathway, whereas DNA-PKcs deficiency is one of the most typical characteristics of GBMs [140].

Notably, the miR-1193/YY1AP1/YY1/FEN1 axis modulates homologous recombination (HR) and MMEJ-mediated DSB repair, offering a compensatory pathway in DNA-PKc-positive cells in which NHEJ-mediated DSB repair is effective [1]. Thus, perturbation of both DSB repair pathways, such as HR/MMEJ and NHEJ, via concurrent blockade of miR-1193 and ablation of DNA-PKcs, is capable of triggering obvious loss of viability in GBM (Table 1) [1]. Also, concurrent blockade of these distinctive DSB repair pathways resulted in stimulation of the ATR/CHK1/p53 axis and consequently facilitated tumor cell apoptosis (Table 1) [1]. Thus, anti-miR-1193 could potentially be used as a single-agent therapy for DNA-PKC-deficient GBM tumors [1].

Interestingly, the silencing of DNA-PKcs and blockade of miR-1193 in GBM is a potential synthetic lethality target for patients with GBM (Table 1) [1]. Furthermore, studies have shown that miRNA inhibition is an essential approach for achieving synthetic lethality in certain tumors with specific genetic deficiencies [141,142]. Chemical modifications of antisense oligonucleotides targeting miRNAs, such as 2′-O-methyl modification, can decrease off-target consequences and considerably increase *in vivo* delivery efficiency [143]. It was further established that a thiol- and cholesterol-conjugated 2′-O-methyl-modified antagonist might be implemented to validate miR-1193 as a potential treatment target for DNA-PKC-deficient GBMs [1]. This emphasizes the significance of the miR-1193/YY1AP1/YY1/FEN1 axis as a potential treatment target for DNA-PKcs-deficient GBMs, with synthetic lethality as the mechanism of action [1].

### 8.1 Future perspective of synthetic lethal effect on glioblastoma

Tumor heterogeneity is one of the most critical hallmarks of the GBM milieu and is characterized by various genetic modulations that allow for the classification of GBM into different molecular subtypes. Notably, GBM has been categorized into four distinctive subtypes, classical, mesenchymal, proneural, and neural, based on the mutational landscape in amalgamation with transcriptional profiles determined via bulk RNA sequencing experiments. Thus, it is possible to design permutations in GBM therapies that can concurrently account for multiple distinct therapeutic effects based on these distinctive GBM subtypes.

Notably, the DDR pathway plays a critical role in the synthetic lethal effect of GBM. Also, the detection of synthetic lethal effects associated with several tumor-specific genotypes led to the permutation of genotype-specific cell-blockade modalities. Permutation of therapeutic modalities may be a way forward in personalizing chemotherapeutic regimens to attain specific effects in GBM. Nevertheless, the combined evaluation of multiple synthetic lethal interactions to identify new combinations of treatment modalities has not been adequately investigated. Specifically, synthetic lethality modalities in biological pathways, such as metabolic reprogramming and oxidative stress in GBM, may be advantageous in GBM therapy. Also, synthetic lethality mechanisms targeting key receptors in GBM may augment the efficiency of therapy.

GBM heterogeneity is accountable for current challenges in synthetic lethal effects because a portion of GBM cells respond to therapy, whereas other portions of the cells are ineffective for therapy. Notably, there are still many difficulties in transforming these synthetic lethal interactions and synergetic therapies into clinical benefits because of the magnitude of specific synthetic lethal effects in GBM. Interestingly, some of the synthetic lethal interactions exhibited adequate significance and biological effects but were incapable of achieving clinical effects because of their minute efficacy in the trials. Furthermore, the DDR pathway, which comprises intricate mechanisms and genes, makes the detection of potential targets problematic.

## 9 Conclusions

The permutation of therapeutic modalities may be the way forward in personalizing chemotherapeutic regimes to attain specific effects in GBM. Currently, PTEN and PI3K; PTEN, PARP, and TMZ; PTEN and IPZ; PTEN and symbiotic macrophage; VRK1 and VRK’, VRK1, TMZ, and olaparib; VRK1, radiation, and DXR; RNAi and siRNA; HDAC1/2 and ClpP; HDAC1/2 and TRAP1; IDH1 and Bcl-xL; Bcl-2/Bcl-xL, and c-MET; CaMKII and GSCs; and DNA-PKcs and miR-1193 constitute potential synthetic lethality targets for the treatment of GBMs. Adequate preclinical and clinical investigations are still needed to establish the most effective and clinically applicable therapeutic modalities for GBM.

## Abbreviations

3′-UTRs : 3′-untranslated regions
AKT: Protein kinase B
ATF4: Activating transcription factor 4
ATM: Ataxia-telangiectasia mutated
ATP: Adenosine triphosphate
ATR: ATM-and Rad3-related
BAF: Barrier to autointegration factor
BAK: BCL-2 antagonist/killer
BAPN: β-Aminopropionitrile
Bax: Bcl-2-associated X protein
BBB: Blood–brain barrier
Bcl-xL: B-cell lymphoma-extra large
BER: Base excision repair
BRCA: Breast cancer gene
Ca^2+^
: Calcium
CaM: calmodulin
CaMKII: Calmodulin (CaM)-dependent protein kinase II
CDK: Cyclin-dependent kinase
ClpP: Caseinolytic protease proteolytic
c-MET: C-mesenchymal–epithelial transition factor
DDR: DNA damage response
DNA-PKcs: DNA-dependent protein kinase catalytic subunit
Doxorubicin: DXR
DSBs: Double-strand breaks
dsRNA: Double-stranded RNA
EGFR: Epidermal growth factor receptor
ERK: Extracellular signal-regulated kinase
FAK: Focal adhesion kinase
FEN1: Flap endonuclease1
GBM : Glioblastoma multiforme
GSCs: GBM stem-like cells
HBC: Hydrazinobenzoylcurcumin
HDAC: Histone deacetylase
HDR: Homology directed repair
HK-1: Hemokinin-1
HR: Homologous recombination
IDH : Isocitrate dehydrogenase
IPZ: Irrepairzepine
IRT: Irradiation/radition
IRT: Radiotherapy
KRAS: Kirsten rat sarcoma viral oncogene homologue
LOX: Lysyl oxidase
MGMT: Neurokinin 1 receptor NK1R,O-6-methylguanine-DNA methyltransferase
mTOR: Rapamycin
NHEJ: Non-homologous end-joining
OCR: Oxygen consumption rate
OXPHOS: Oxidative phosphorylation
PARP: Poly(ADP-ribose) polymerase
PARPi: PARP inhibitor
PI3K: Phosphoinositide 3-kinases
PTD: Peptide transduction delivery domain
PTD-DRBD: PTD fused to a dsRNA-binding domain
PTEN: Phosphatase and tension homologue
PYK: Proline-rich tyrosine kinase
RNAi: RNA interference
RPA: Replication protein A
siRNA: Short interfering RNA
STAT: Signal transducer and activator of transcription
TAMs: Tumor-associated microphages
TAT: Transactivator of transcription
TME: Tumor microenvironment
TMZ: Temozolomide
VRKs: Vaccinia-related kinase
YAP: Yes-associate protein
